# Dynamics of growth zone patterning in the milkweed bug *Oncopeltus fasciatus*

**DOI:** 10.1242/dev.142091

**Published:** 2017-05-15

**Authors:** Tzach Auman, Barbara M. I. Vreede, Aryeh Weiss, Susan D. Hester, Terri A. Williams, Lisa M. Nagy, Ariel D. Chipman

**Affiliations:** 1 Department of Ecology, Evolution and Behavior, The Alexander Silberman Institute of Life Sciences, The Hebrew University of Jerusalem, Edmond J. Safra Campus, Givat Ram 91904, Jerusalem, Israel; 2 Faculty of Engineering andThe Bar-Ilan Institute of Nanotechnology & Advanced Materials, Bar Ilan University, Ramat Gan 52900, Israel; 3 Bio-Imaging Unit, The Alexander Silberman Institute of Life Sciences, The Hebrew University of Jerusalem, Edmond J. Safra Campus, Givat Ram 91904, Jerusalem, Israel; 4 Molecular and Cellular Biology Department, University of Arizona, Tucson, AZ 85721, USA; 5 Biology Department, Trinity College, Hartford, CT 06106, USA

**Keywords:** Segmentation, Arthropod, Growth zone, Cell division, Terminal addition

## Abstract

We describe the dynamic process of abdominal segment generation in the milkweed bug *Oncopeltus fasciatus*. We present detailed morphological measurements of the growing germband throughout segmentation. Our data are complemented by cell division profiles and expression patterns of key genes, including *invected* and *even-skipped* as markers for different stages of segment formation. We describe morphological and mechanistic changes in the growth zone and in nascent segments during the generation of individual segments and throughout segmentation, and examine the relative contribution of newly formed versus existing tissue to segment formation. Although abdominal segment addition is primarily generated through the rearrangement of a pool of undifferentiated cells, there is nonetheless proliferation in the posterior. By correlating proliferation with gene expression in the growth zone, we propose a model for growth zone dynamics during segmentation in which the growth zone is functionally subdivided into two distinct regions: a posterior region devoted to a slow rate of growth among undifferentiated cells, and an anterior region in which segmental differentiation is initiated and proliferation inhibited.

## Abstract (precis)

**Summary:** A detailed analysis of posterior segment addition in an insect reveals that the growth zone is divided into two functional domains responsible for growth and differentiation.

## INTRODUCTION

A segmented body plan is a fundamental feature of arthropods. Nevertheless, the mode of segment determination varies considerably among different taxa, even within insects (Lynch et al., 2012). The fruit fly *Drosophila melanogaster* has been used as the main model organism for understanding the mechanisms of segmentation. However, with all of its advantages, *Drosophila* exhibits a derived form of embryonic development, making it a poor representative of more common and widespread types of arthropod segmentation (Davis and Patel, 2002; Mito et al., 2010; Peel et al., 2005).

In *Drosophila*, all body segments are defined almost simultaneously via a process starting in the early syncytial blastoderm stage of development and mediated by a cascade of interacting transcription factors (Hartenstein and Chipman, 2015; Lawrence, 1992; Nüsslein-Volhard and Wieschaus, 1980). This mode of development is referred to as ʻlong-germ' development. By contrast, the development of many basally branching insects is characterized by a mode of segmentation known as ʻshort-germ' (Davis and Patel, 2002; Krause, 1939; Liu and Kaufman, 2005b; Sander, 1976) or sequential segmentation. In this mode of development, only some segments form in the blastoderm stage. These include the head segments and may include, depending on the species, some or all of the thoracic segments. The remaining segments (some or all of the thoracic segments as well as the abdominal segments) are defined sequentially, one segment after the other from anterior to posterior, from a cellularized region at the posterior of the embryo. This region is referred to as the ʻgrowth zone' or as the ʻsegment addition zone' (Janssen et al., 2010). Sequential segmentation in insects is believed to reflect the ancestral mode of segmentation in arthropods (Peel et al., 2005; Stahi and Chipman, 2016).

While the details of simultaneous segmentation have been studied extensively in *Drosophila*, much less is known about the mechanisms of sequential segmentation. Specifically, many questions remain regarding the cellular aspects of this process, which takes place in a very different tissue environment compared with *Drosophila* (Peel et al., 2005). The role of cell proliferation and migration in the formation of segments from the growth zone varies among species and modes of segmentation (Beermann et al., 2011; Chipman, 2008; Copf et al., 2004; McGregor et al., 2009; Nakamoto et al., 2015; Oberhofer et al., 2014; Peel et al., 2005; Ten Tusscher, 2013). Whereas some require intensive cell proliferation [e.g. malacostracan crustaceans (Dohle and Scholtz, 1988; Scholtz, 1992; Wolff and Scholtz, 2002)], others, such as the centipede *Strigamia maritima*, seem to rely mainly on a pre-established pool of cells (Brena and Akam, 2013; Chipman et al., 2004b). The relative contribution of these two sources of cells to newly formed segments among different arthropods is unknown.

The embryology of the milkweed bug *Oncopeltus fasciatus*, a short-germ hemipteran, has been described using classical techniques (Butt, 1949) and it is now re-emerging as a model for basal insect development. Its ease of rearing, availability of molecular tools, and recently sequenced genome make *Oncopeltus* an appealing system. In the development of *Oncopeltus*, all head, gnathal and thoracic segments are specified during the blastoderm stage (Ben-David and Chipman, 2010; Birkan et al., 2011; Liu and Kaufman, 2005b; Stahi and Chipman, 2016), in a manner resembling segmentation in *Drosophila.* By contrast, formation of abdominal segments starts *de novo* from a pool of undifferentiated cells in the growth zone. The growth zone is formed by the migration and proliferation of cells in the posterior blastoderm (Liu and Kaufman, 2004). These cells first form a small invagination in the posterior pole, moving inwards as the formation of the primordial germband occurs. By the time the germband has formed, ingressed cells include those fated to become the head and the thoracic segments and an undetermined region of cells that will undergo elongation and sequential segmentation to give rise to the abdominal segments.

In this work, we describe the dynamic process of segment generation in detail, by combining a morphometric analysis of carefully timed specimens with spatial and temporal patterns of cell division and gene expression. We show that the growth zone is functionally subdivided into two separate regions: a posterior region of undifferentiated cells devoted to growth – both through contributions of pre-existing cells and through cell division – and a region of reduced cell division devoted to initiating the specification of segments. Each region correlates with specific expression patterns of segmentation genes. We find that during the addition of a single segment, the growth zone undergoes dynamic changes in shape. We also observe significant variability in the size of the growth zone between individual *Oncopeltus* embryos, suggesting that the mechanisms that regulate segment addition from the posterior are robust to variations in size.

## RESULTS

### Dynamics of the growth zone and newly formed segments

**Fig. 1.**
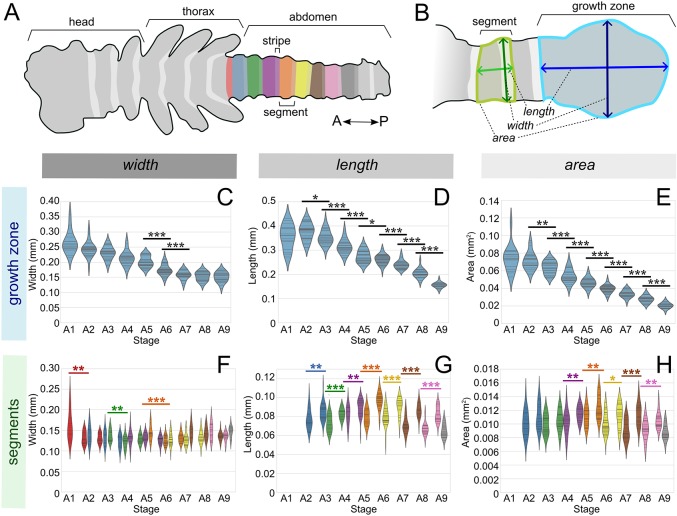
**Measurements made on the growth zone and segments.** (A) Illustration of an *Oncopeltus* *fasciatus* embryo after segmentation is complete. Abdominal segments are color-coded, with the posterior *inv* stripe of each segment in a lighter shade. (B) The growth zone and newly formed segments, indicating the measurements made. (C-E) Violin plots representing the distribution of measurements on the growth zone by developmental stage (number of *inv* stripes). Pairwise one-way ANOVAs were performed to assess statistically significant changes in dimensions from one stage to the next. *P<0.05, **P<0.01, ***P<0.001. (F-H) Violin plots representing the distribution of measurements on the two most recently formed segments, and the three most recently formed *inv* stripes, by developmental stage (number of *inv* stripes). The colors correspond to the segment colors in A. Pairwise one-way ANOVAs were performed to assess statistically significant changes in dimensions of the same segment from one stage to the next. *P<0.05, **P<0.01, ***P<0.001 (colors correspond to the segment in question). In F, asterisks above the plot concern the first pair of measurements of a segment (e.g. stage A1 versus A2 for segment 1).

We measured various morphological parameters in the growth zone and recently formed segments over time (Fig. 1A,B, Fig. S1, Tables S1-S3). Throughout the germband stage, the growth zone extends posteriorly, while new segments emerge from its anterior end. The size of the growth zone – defined as the area from the posterior of the embryo to the posteriormost stripe of the segment polarity gene *invected* (*inv*) – decreases gradually in all dimensions (Fig. 1C-E). To confirm that the decrease in size is not due to cell death we carried out anti-caspase staining, and found no notable pattern of apoptosis in the growth zone during these stages (Fig. S2).

Conversely, once a segment has been formed (as defined by the expression of a new *inv* stripe), it continues to grow in size. Importantly, growth within a segment at this time occurs solely along the anterior-posterior axis (segment length, Fig. 1G). By contrast, segment width, as measured along the *inv* stripe, varies little throughout the segmentation of the abdomen (Fig. 1F). With the exception of the small decrease in width of the seventh abdominal stripe (establishing the sixth abdominal segment) between stages A8 and A9 (Fig. 1F, *P*<0.01), segment width is the most constant dimension we measured. Conversely, segment length increased significantly for every segment following its formation, and segment area increased significantly for all except the first and second segments (Fig. 1G,H). We note that although all of the trends detailed above are clear, there is a fair amount of variability within each parameter.

### Growth and segmentation

**Fig. 2.**
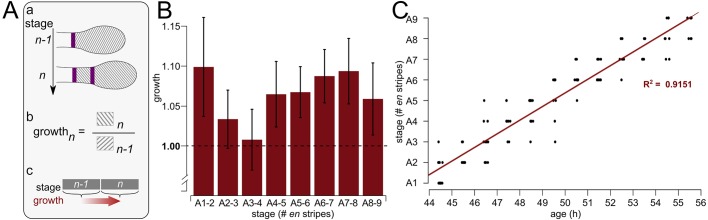
**Growth and segmentation.** (A) (a) The areas used for the calculation of growth. (b) Growth at stage *n* was measured by dividing the combined area of the growth zone and the most recently formed segment by the average area of the growth zone in stage *n*−1. (c) Owing to the way that growth is calculated (i.e. using the averages of all individuals in the same stage), the developmental time for which growth is calculated is offset with respect to the segmental stage. The schematic shows how stages used on the *y*-axis for C and the *x*-axis for B relate to each other. (B) Growth calculated as shown in A, using measurement averages. Error bars indicate the (propagated) s.e.m. (C) Staged embryos collected in 60-min windows (*n*=123), from 44-45 to 55-56 hAEL, as well as the linear model fitting these data (red line).

During development the growth zone gradually decreases in size due to the formation of new segments. However, while new segments are formed the growth zone itself is in fact growing, but not at a rate that compensates for the loss of area due to segment formation, with the exception of the transition between stages A1 and A2 (Fig. 1E). To assess growth during a single stage, the average area of the growth zone at the current stage plus the area of the most recently formed segment was divided by the average area of the growth zone in the previous stage (Fig. 2A). This value includes a simplification, as it assumes that the thickness of the growth zone and the newly formed segment are equal and that the thickness of both is roughly uniform over time. Although there might be a change in thickness in the transition from growth zone to segmental tissue, this would cause a slight underestimation or overestimation of the growth rate, but should not affect the pattern observed. In this calculation, a value of 1 indicates that the size of the new segment is equal to the area lost by the growth zone and that no additional growth has taken place. Conversely, a value >1 indicates that growth took place in the growth zone and/or latest segment during this stage.

Calculated this way, our data show that, per stage, the growth zone increases in size between 0.9±3.7% (stage A3-A4) and 9.8±6.1% (stage A1-A2) (Fig. 2B; note that error bars represent propagated standard error of a calculation and not the distribution of a direct measurement). Although growth rates appear to differ from one stage to the next, these differences are not significant.

Nevertheless, this possibly discontinuous growth relative to segment number, in particular the change in growth rate from segment A1-A2 to A3-A4, raises the question of how growth relates to segmentation rate. Specifically, we wanted to establish whether abdominal segmentation is a linear process or if the rate of segmentation changes between stages. Our ability to resolve this question is complicated by the combination of the reproductive biology of *Oncopeltus* and our method of egg collection. Because eggs are laid in large clutches, at a rate of approximately one egg every 1-2 min, a single clutch might constitute a large proportion of the eggs sampled in a time window. This would inadvertently bias the birthdate distribution within the sample time window by essentially synchronizing a large part of the sample. Thus, we collected a second data set in which clutches were explicitly broken up and randomized over different time windows. The data show a linear rate of segmentation (R^2^=0.9151), with a new abdominal segment forming every 1.5 h (Fig. 2C). No evidence was found to indicate a deviation from the linear segmentation rate (Chi-squared test, *P*=0.6738; see the Materials and Methods).

### Gene expression in the growth zone

We then asked how the morphology of the segmenting germband correlates with molecular processes. To address this, we followed the expression of four genes with documented roles in sequential segmentation in insects.

**Fig. 3.**
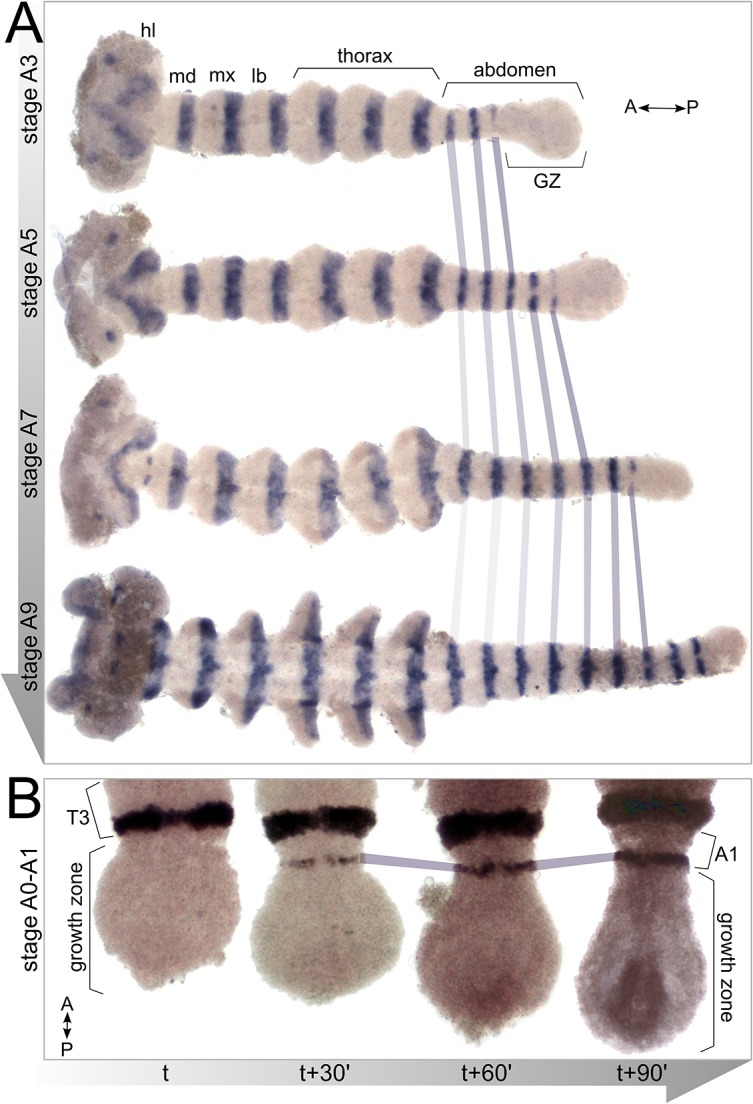
**Changes in the growth zone throughout the segmentation process.** (A) Embryos of increasing age (stages A3-A9) stained for expression of *inv*. Note the gradual decrease in growth zone size as segmentation proceeds. (B) Early embryos in stage A0-A1 (during formation of the first abdominal segment). Embryos from a single clutch fixed in 30-min intervals demonstrate changes in growth zone dimensions during the formation of a single segment. The growth zone begins round, gradually elongating and assuming a teardrop shape as the new segment slowly buds from the anterior growth zone. hl, head lobe; md, mandibular segment; mx, maxillary segment; lb, labial segment; T3, third thoracic segment; A1, first abdominal segment.

*inv* mRNA is expressed, as expected, in the posterior of each molecularly defined segment. *inv* is a paralog of *engrailed* (*en*) and has very high sequence identity and, in some species, identical expression (Campbell and Caveney, 1989; Peel et al., 2006; Peterson et al., 1998). We and others use the *en/inv* expression pattern to define the border between the segmented germband and the unsegmented growth zone (Fig. 3).

Fig. 3A shows a sequence of *inv* stained embryos from the beginning of posterior segmentation (stage A1) up to determination of the ninth abdominal segment (stage A9, at which point our analysis ended). Fig. 3B shows the dynamics of *inv* expression over the formation of a single segment (in this case, the first abdominal segment). For this analysis, a single clutch was collected within 30 min and individual embryos were sampled from this clutch every 30 min (note that there is some variability in the age of the embryos within a clutch, so the timing is not precise). The expression of an individual segmental *inv* stripe matures over the duration of 1 h, appearing initially as a thin stripe that is discontinuous along the ventral midline, gradually thickening and maturing into a continuous stripe. Although we did not carry out high temporal resolution analysis for the formation of other segments, the variation observed in the posteriormost stripe within our other samples indicates that this sequence of events is typical of other abdominal segments.

The timecourse in Fig. 3B also shows that the shape of the growth zone changes dramatically during the formation of a new segment. The growth zone starts out round and elongates to a teardrop shape as the *inv* stripe is consolidated. Although we have not followed the shape change during the addition of more posterior stripes, we suggest that this change in shape is indicative of a cyclic process of cells rearranging in the posterior to form the next segment, and that this process might be occurring during the formation of the other segments as well.

**Fig. 4.**
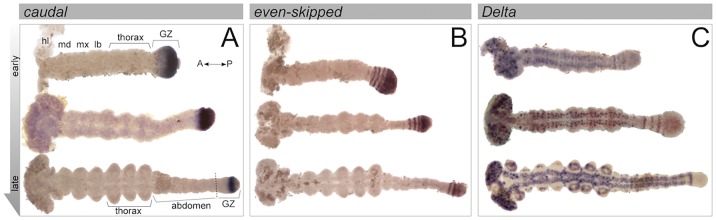
**Developmental gene expression patterns during germband segmentation.** Expression pattern of *cad* (A), *eve* (B) and *Dl* (C) mRNA at different developmental stages, from the earliest germband (∼40 hAEL) to the final stages of abdominal segmentation (∼55 hAEL). (A) *cad* is stably expressed in the posterior growth zone throughout germband segmentation. (B) *eve* displays a more complex expression pattern, in which the entire posterior growth zone expresses *eve*, yet in the anterior growth zone *eve* is expressed in a dynamic striped pattern. (C) *Dl* is expressed in the anterior growth zone in a varying number of stripes, with no expression in the posterior growth zone. More anteriorly, *Dl* is expressed in the nervous system, seen as two mediolateral lines of punctate expression extending posterior from the head lobes to (but not including) the growth zone. GZ, growth zone; hl, head lobe; md, mandibular segment; mx, maxillary segment; lb, labial segment.

*caudal* (*cad*) mRNA is expressed in a stable and uniform manner in the posterior of the growth zone throughout development (Fig. 4A). Expression is strongest in the posterior of the growth zone, and diminishes in a gradient towards the anterior of the growth zone, from which it is absent. There is almost no change in the extent or level of *cad* expression in the growth zone throughout the segmentation process. After all segments have formed, expression clears slightly from the very posterior of the growth zone, perhaps indicating that the process of segmentation is completed.

*even-skipped* (*eve*) mRNA expression (Fig. 4B) is characterized by two distinct areas. In the posterior growth zone *eve* expression is uniform and stable throughout germband elongation and segmentation. By contrast, *eve* expression in the anterior growth zone displays a striped pattern. The number of stripes is variable and dynamic, with three to four stripes in the earlier stages of segmentation and only two or three stripes at later stages. The expression of *eve* in *Oncopeltus* has been described in detail by Liu and Kaufman (2005a).

*Delta* (*Dl*) mRNA has a more complex expression pattern (Fig. 4C). It is expressed in two distinct domains. The first is a speckled pattern marking pro-neural tissue in the head lobes, and continuing along the segmented germband in two mediolateral rows of pro-neural cells, as described previously in other arthropods (Chipman and Stollewerk, 2006; Eriksson et al., 2013; Kainz et al., 2011; Stollewerk and Chipman, 2006). More relevant to segmentation, *Dl* is expressed in stripes in the anterior growth zone. Two to three stripes are present in the anterior growth zone in early stages of segmentation, with one or two stripes at late stages of the process. The stripes vary in strength of expression, and the position of the strongest stripe is variable between embryos collected within the same 2 h time window. Closer examination reveals that there is sometimes an overlap between the segmental and pro-neural patterns, with stronger expression in lateral spots within the segmental stripes. Notably, there is almost no detectable *Dl* expression in the posterior growth zone during segmentation.

### Cell proliferation

**Fig. 5.**
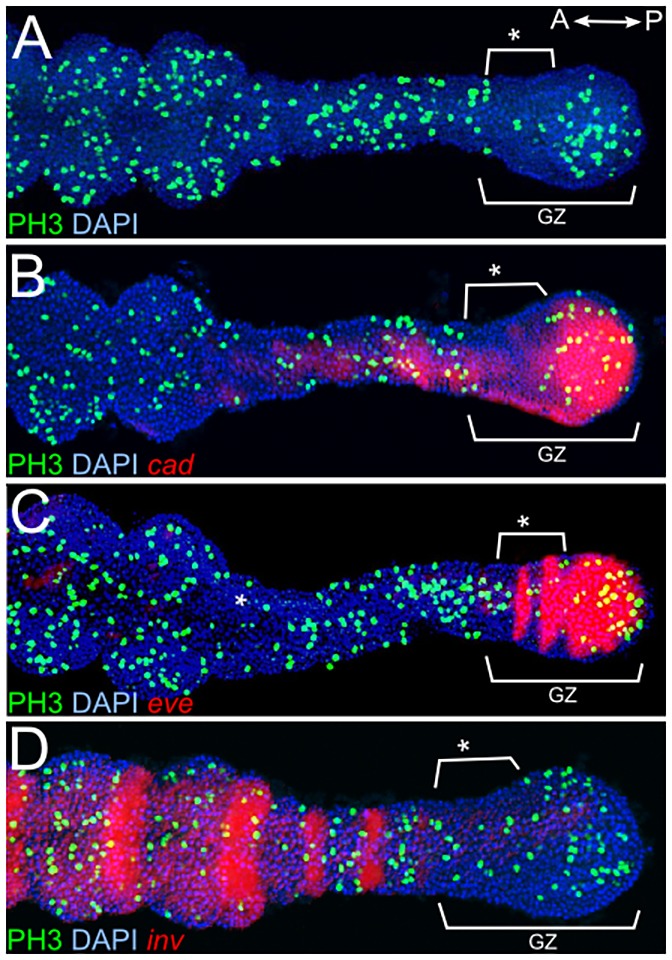
**Cell division and gene expression in the growth zone.** Double stainings of anti-PH3 (green), as an indicator of cell division, with *in situ* hybridization for segmentation genes (red pseudocolor, detected using brightfield). DAPI is used as nuclear counterstain (blue). The precise age of the embryos is not known, but they are all towards the end of posterior segmentation (∼50 hAEL). Brackets labeled with an asterisk mark a gap in cell proliferation in the anterior growth zone. (A) Embryo stained with anti-PH3 and DAPI without *in situ* staining. A gap in cell proliferation (asterisk) is noticeable in the anterior growth zone. (B) *cad* staining correlates with an area with increased PH3^+^ cells. Note that the anterior red staining is an artifact of the image merge process and is not seen in single-stained embryos (compare with Fig. 4A). (C) *eve* in the posterior overlaps with *cad* expression. The striped expression pattern of *eve* corresponds to an area with decreased PH3 staining. (D) *inv* marks the anterior border of the growth zone and the boundary between high PH3 staining (anteriorly) and low PH3 staining (posteriorly).

**Fig. 6.**
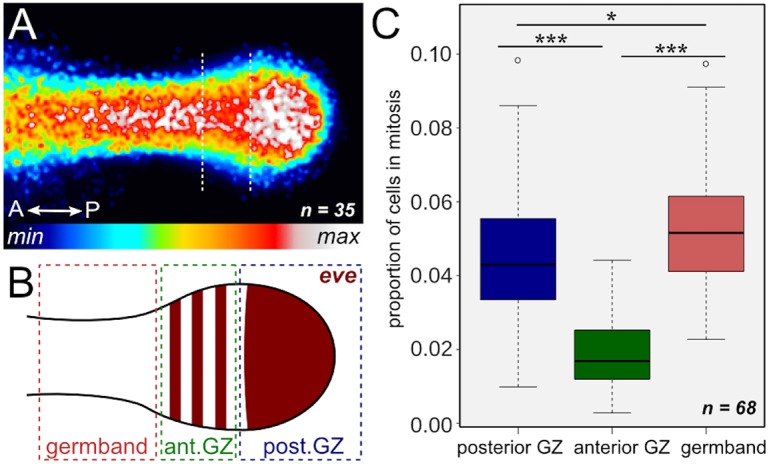
**Cell proliferation in different areas of the germband.** (A) Heat map of cell proliferation in the germband. Merged image of 35 PH3-stained germbands in various stages, aged from 46-54 hAEL, aligned at the widest part of the growth zone, translated to a look-up table (legend beneath the panel). The dotted lines delineate the zone of low proliferation. (B) Illustration of *eve* expression, indicating the different areas for which cell proliferation was calculated in C. (C) Proportion of cells in mitosis in each area (see B), calculated from the number of PH3^+^ cells among total cells (DAPI staining). Error bars indicate s.e.m. *P<0.05, ***P<0.001 (ANOVA).

Labeling for phosphorylated histone 3 (PH3) to mark cells in mitosis uncovers a simple, yet striking, pattern (Fig. 5A, Fig. 6A) in which cell proliferation is detected in the posterior part of the growth zone, followed by a gap or ʻwindow' of variable size and appearance in the anterior growth zone (Fig. 5, asterisk), where there is clearly decreased cell proliferation. An increase in relative cell proliferation is detected anterior to this window in the segmented germband. No similar gap is observed in other embryonic regions.

In order to better describe the borders of this domain of decreased PH3, and possibly its role in the segmentation process, the PH3 labeling was repeated in combination with *in situ* hybridization for the genes detailed above. The most notable link between PH3 staining and gene expression is the overlap between the posterior proliferative zone and the expression of *cad* and the posterior expression region of *eve* (Fig. 5B,C). The domain of reduced proliferation correlates with the region of striped *eve* expression, and expression of *inv* is always anterior to this domain (Fig. 5D). The borders of *Dl* expression do not correlate consistently with the borders of this window (data not shown). These correlations are consistent throughout the entire process of abdominal segmentation.

To further quantify the distribution of PH3-positive cells with *eve* mRNA expression, we analyzed 68 embryos ranging from 44-56 hours after egg laying (hAEL) labeled for both *eve* and PH3. We chose the expression pattern of *eve* for delimiting zones for detailed analysis of cell proliferation (Fig. 6B) because it provides a pattern defining three primary regions of interest: (1) the posterior growth zone, defined by a solid staining in the most posterior part of the embryo; (2) the anterior growth zone [roughly equivalent to the pre-segmental region, as in Schröder et al. (2008)], defined by a striped expression pattern; and (3) the segmented germband, which extends anterior from the anteriormost *eve* stripe.

Using a custom-designed macro, we scored the total number of cells in each of these regions (counting nuclei labeled with DAPI), as well as the number of cells undergoing mitosis (counting cells positive for PH3). The ratio between these counts gives us the fraction of cells that are undergoing mitosis in the different regions (Fig. 6C). Indeed, as detected visually, the relative number of proliferating cells in the anterior growth zone is significantly lower (ANOVA, *P*<0.001) than in the posterior growth zone. The fraction of proliferating cells in the segmented germband is also significantly higher (ANOVA, *P*<0.001) than in the anterior growth zone, as well as in the posterior growth zone, although this difference is marginal (but nonetheless significant: ANOVA, *P*<0.05). This pattern is consistent throughout abdominal segmentation (Fig. S3, Table S4).

## DISCUSSION

The development of the abdominal segments in the hemipteran *Oncopeltus fasciatus* occurs through the sequential addition of segments from a posterior growth zone. While there is a growing body of data on sequential segmentation in other arthropods (Brena and Akam, 2013; Chipman and Akam, 2008; Choe et al., 2006; Copf et al., 2004; Damen et al., 2000; Janssen et al., 2010; Kainz et al., 2011; Lynch et al., 2012; McGregor et al., 2009; Nakamoto et al., 2015; Olesnicky et al., 2006; Sarrazin et al., 2012; Schoppmeier and Damen, 2005; Williams et al., 2012), we are the first to combine carefully timed morphometric measurements with gene expression patterns and measurements of cell division. This combined approach allows us a more precise understanding of the dynamic process of segment addition in *Oncopeltus*. In this work we have focused on posterior segmentation, aiming to provide novel insights into the basic morphological dynamics during germband elongation and segmentation in the insect embryo, and into the involvement of cell proliferation and key developmental genes in this process.

### Morphological changes in the growth zone

The most obvious result from our morphological analysis of the growth zone is its steady decrease in size throughout segment generation, as tissue leaves the growth zone to contribute to the nascent segments. This is hardly surprising and, although it has rarely been quantified, it is a well-known aspect of the process of segmentation. These results mirror the findings of Nakamoto et al. (2015) in the embryo of the red flour beetle *Tribolium castaneum*. The decrease in size in *Oncopeltus* is manifested mostly in the length of the growth zone and, to a lesser extent, in its width, leading to a constant decrease in area. Once new segments are defined molecularly, their width changes very little throughout the segmentation process. Meanwhile, their length and area increase slightly, indicating that there is additional growth after segment formation, as would be expected from the observed cell proliferation in the nascent segment.

### Variability

One of the intriguing points arising from our measurements is the large variance in growth zone and segment size and shape. Since the mean value of segment width changes little, if at all, over development (Fig. 1F), its variability at different stages is a good indication of the overall variation that exists between individuals (see Appendix S1). Variation in this one-dimensional parameter (i.e. width of the penultimate stripe) spanned an up to twofold difference between the smallest and largest embryo measured. Confounding factors in this observation could be experimental, such as measurement or mounting errors. To account for measurement errors, all photos were taken at a standard magnification and all measurements were repeated three times and averaged. Mounting differences between slides were found to account for some of the variance in our measurements (∼21.5%; see Appendix S1), leaving a conservative 52% increase in this parameter between the smallest and largest animals. Some of this variability is no doubt related to the known variability in egg size in insects (Houchmandzadeh et al., 2002); however, we did not measure egg size prior to dissection of the germband. The variability suggests that the segmenting embryo is robust to large changes in the surface area of the growth zone.

### Short-term changes in shape in the growth zone

In Fig. 3B, we show dramatic changes in growth zone shape that occur during the formation of a single segment. These changes demonstrate that some variance is due to the substages of segment formation, and show that the shape of the growth zone and of the nascent segments is dynamic during the addition of each segment. Although the cellular mechanisms responsible for this more rapid shape change were not a part of this study, we speculate that coordinated changes of cell shape and or cell rearrangements driven by actin-myosin contractions might play a role. Unraveling the complexities of the subphases of segment generation requires much higher temporal resolution than we have been able to achieve in the current study and must await future work.

### Rate of segment generation

Although a reiterative process, abdominal segmentation may not occur at a steady rate (Fig. 2C). In *Oncopeltus*, we show that the segmentation rate is linear (Fig. 2C). By contrast, a non-linear segmentation rate has been shown in *Tribolium* (Nakamoto et al., 2015). In *Tribolium*, sequential segment addition includes all the post-mandibular segments and the change in segmentation rate occurred during the transition from thoracic to abdominal segment addition. Because the change in segmentation rate correlated with a change in the behavior of marked blastoderm clones, Nakamoto et al. (2015) hypothesize that the two might be linked, and thus change in a coordinated fashion during the transition from thoracic to abdominal segmentation. In *Oncopeltus*, the constant segmentation rate is observed within the production of a single body tagma.

### The source of segmental tissue

There is some debate over whether there is any growth (generation of new tissue) in the growth zone, or whether all of the tissue that contributes to new segments is present from its origin (Chipman, 2008; Peel et al., 2005). Our data show that most of the tissue in newly formed segments in *Oncopeltus* is derived from existing growth zone tissue. However, there is a certain contribution of cell proliferation to this process, as the tissue recruited to the new segment (in most cases) is greater than the decrease in size of the growth zone, and cells undergoing proliferation in the posterior of the growth zone are detected during all stages analyzed.

Most of the proliferation occurs at the posterior of the growth zone, at the point in the growth zone most distant from where the nascent segments form (Fig. 6A, Fig. S3). Conversely, tissue from the anterior part of the growth zone contributes to the formation of new segments. While the growth zone diminishes in size with the formation of each segment, it is replenished to a certain extent by cell proliferation in the posterior.

### A functional model for the arthropod growth zone

**Fig. 7.**
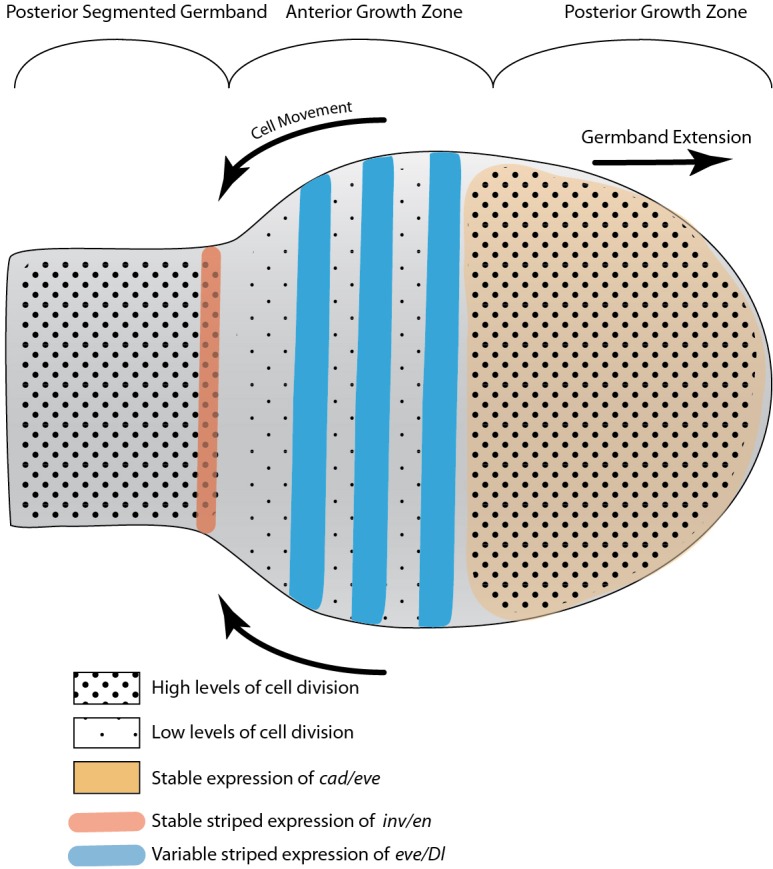
**Model of the *Oncopeltus* growth zone.** The segmentation process takes place over three distinct domains. The posterior growth zone is characterized by the expression of *cad* and the stable expression of *eve* and probably other pair-rule gene orthologs (jointly indicated in yellow). It is also characterized by a relatively high level of cell division (densely packed large dots). The anterior growth zone is characterized by the dynamic expression of pair-rule gene orthologs; in some other arthropods Notch pathway ligands have a similar expression domain (jointly indicated by blue stripes). Cell division levels are significantly lower (sparsely distributed small dots) than in the domains anterior and posterior to it. Cell movements in this domain lead to the constriction of the growth zone and to the extension of the posterior growth zone posteriorly. The dynamic cyclical expression of genes in this domain leads to the sequential sequestering of the anteriormost tissue into the segmented germband. The posterior of the germband is defined by the expression of *inv* (red stripe). Cell division levels in this area are higher (densely packed large dots) than in the anterior growth zone.

Our data allow us to formulate a generalized model for *Oncopeltus* (Fig. 7) and to use it as a basis of evolutionary comparison among arthropods. This model is consistent with partial data from several other arthropod species (Brena and Akam, 2012, 2013; Chipman and Akam, 2008; Nakamoto et al., 2015; Schröder et al., 2008; Williams et al., 2012).

We see the segmentation process taking place over three distinct embryonic domains. The posterior growth zone contains undifferentiated cells. This domain is characterized by the expression of *cad* and by stable expression of *eve.* At the cellular level, proliferative activity is detectable in this domain throughout segmentation in *Oncopeltus*, with a similar result being reported for *Tribolium* (Sarrazin et al., 2012). Uniform expression of both *cad* and *eve* has been demonstrated in the posterior growth zone of many arthropods (Brown et al., 1997; Chipman and Akam, 2008; Copf et al., 2003; Dearden and Akam, 2001; El-Sherif et al., 2014; Hughes and Kaufman, 2002; Mito et al., 2007; Patel et al., 1994; Shinmyo et al., 2005). We suggest that *cad*, and potentially *eve*, are responsible for maintaining the cells of the posterior growth zone in an undifferentiated state, and that this is a general feature of arthropod posterior growth zones. Wnt signaling is upstream of *cad* in several arthropods (Chesebro et al., 2013; McGregor et al., 2009; Shinmyo et al., 2005) and might be the initiator of growth zone function.

The anterior growth zone is where cells undergo a series of specification events leading up to their recruitment into nascent segments. This domain is characterized by the expression of genes in a dynamic pattern. In our results for *Oncopeltus*, and in the equivalent domain of other arthropods, these include orthologs of *Drosophila* pair-rule genes and, in some cases, also Notch pathway genes (Brena and Akam, 2013; Chipman and Akam, 2008; Choe et al., 2006; Damen et al., 2000; El-Sherif et al., 2014; Eriksson et al., 2013; Mito et al., 2007, 2011; Patel et al., 1994; Pueyo et al., 2008; Stollewerk et al., 2003). In *Tribolium*, a traveling wave underlies the dynamic expression pattern of the pair-rule genes, and the expression data in other species are also consistent with this idea (Chipman and Akam, 2008; El-Sherif et al., 2012; Pueyo et al., 2008; Schoppmeier and Damen, 2005). We suggest that this domain is equivalent to the ʻtransition zone' of the centipede *Strigamia maritima* (Chipman et al., 2004a) and the pre-segmental region (PSR) of *Tribolium castaneum* as defined by Schröder et al. (2008). Using carefully timed embryos in a combined analysis of gene expression and patterns of cell division, we found that the most conspicuous characteristic of this domain in *Oncopeltus* is a significantly decreased level of cell proliferation. We find evidence for a similar correlation of proliferative activity in the fairy shrimp, *Thamnocephalus*: cells of the anterior growth zone do not undergo DNA synthesis, whereas those in the posterior growth zone do (as indicated by EdU incorporation; T.A.W., unpublished). We predict that a similar correlation between reduction in cell division and the onset of segmental specification will be found in other arthropods.

Cells of the anterior growth zone are sequestered into the third domain, namely the posterior segmented germband, where they start expressing segment polarity genes and differentiate into segmental tissue with distinct fates. Based on what has been shown in *Tribolium*, this sequestration most likely includes a significant contribution of cell movement (Benton et al., 2013; Sarrazin et al., 2012), probably cell intercalation through convergent extension, but we have not attempted to follow such movements in the current analysis. In this domain, cell proliferation and growth continue. The boundary between the posterior segmented germband and the anterior growth zone is defined by the expression of *inv/en.*

### Concluding remarks

The term ʻgrowth zone' has fallen out of favor in recent years since this term had traditionally been assumed to refer to a region of high proliferative activity used to generate a continual supply of cells for segment generation. More recent work has demonstrated the diversity of patterns of cellular activity within the growth zones of sequentially segmenting arthropods (Brena and Akam, 2013; Chipman, 2008; Chipman et al., 2004b; Copf et al., 2004; Dearden and Akam, 2001; Dohle and Scholtz, 1988; El-Sherif et al., 2012; Kainz et al., 2011; Nakamoto et al., 2015; Olesnicky et al., 2006; Pueyo et al., 2008; Scholtz, 1992; Williams et al., 2012), ranging from species that rely heavily on posterior proliferative activity to those that rely more extensively on cellular rearrangements, to all manner of variation between these two extremes. We have shown that there is growth through cell proliferation as well as contributions from pre-existing cells in the growth zone of the milkweed bug *Oncopeltus fasciatus.* We argue for retaining ʻgrowth zone' as a generally understood term for the area from which the germband grows – albeit using a diversity of cellular mechanisms.

Our analysis provides a highly detailed description of the processes involved in posterior segmentation by characterizing the cellular domain in which it arises and by linking cell division to the expression of segmental regulators. Posterior segmentation is a defining feature of arthropods and clearly appeared early in their evolutionary history. A better comparative understanding of how known regulators operate within a diverse array of cellular contexts will contribute to our insight into the evolution of the arthropod body plan.

## MATERIALS AND METHODS

### Embryo preparation

Methods for the embryology of *Oncopeltus* (egg collection, fixation, dissection, *in situ* hybridization and imaging) are as previously described (Ben-David and Chipman, 2010). Unless otherwise noted, embryos were collected in 1 h and 2 h windows and placed in a 25°C incubator until fixation at the age of interest. In some cases, collections consisted of part of a clutch that was in the process of being laid.

### Gene cloning and probe preparation

Two of the four probes used in this study were for genes cloned previously. The gene we refer to as *invected* (*inv*) was originally identified as *engrailed* and has appeared as such in published papers (Angelini and Kaufman, 2005; Erezyilmaz et al., 2009; Liu and Patel, 2010; Weisbrod et al., 2013). However, a re-analysis of this sequence following the sequencing of the full genome of *Oncopeltus* revealed that it is in fact the *engrailed* paralog *invected* (Peel et al., 2006). This sequence was extended using primers designed from genomic sequence to produce a probe of 873 bp.

The sequence for *even-skipped* (*eve*) is based on Liu and Kaufman (2005a), and was extended using primers based on the full genomic sequence to produce a probe of 770 bp. Cloning of *caudal* (*cad*) employed gene-specific primers designed according to an unpublished transcriptome, to produce a probe of 513 bp, and later verified using genomic data. *Delta* (*Dl*) was originally cloned through degenerate PCR, then recloned using specific primers based on genomic sequence to produce a probe of 712 bp.

Probes were prepared with digoxigenin-labeled UTPs (Roche) using the DIG RNA Labeling Kit (Roche), with linearized T-easy plasmids (Promega) containing the target sequence as template (Ben-David and Chipman, 2010). The primers used for the probes are listed in Table S5.

### *In situ* hybridization and antibody staining

*In situ* hybridization was carried out as described previously (Ben-David and Chipman, 2010) and developed using BM-Purple (Roche).

In double stainings with *in situ* hybridization, anti-phosphorylated histone H3 (PH3) antibody (1:500; Abcam, ab14955) was added simultaneously with the alkaline phosphatase-conjugated anti-DIG antibody (1:4000; Roche) in an overnight incubation at 4°C. Three to five washes were performed at room temperature the following day, and after a 30 min secondary block in 10% normal horse serum (Vector Labs) or normal goat serum (Thermo Scientific), the secondary antibody (1:200; Alexa 448, anti-mouse, Invitrogen) was added for a 2 h incubation in the dark. After three to five washes, the standard *in* *situ* hybridization protocol was followed.

### Growth zone measurements

Growth zone measurements were performed manually on captured images of embryos stained for the segment polarity gene *inv*, using a Fiji (Schindelin et al., 2012) macro designed to collect and organize the data. The dimensions that were measured included length, width and area of the growth zone and of the two posteriormost segments (Fig. 1). The width of the growth zone was measured at its widest point. The segment widths were measured on the *inv* stripes. Each dimension was measured three times, and the average was used as a single data point. The areas of interest were measured in 235 embryos, covering all stages of abdominal segmentation (for A1-A9, *n*=16, 27, 26, 24, 27, 43, 26, 23, 23, respectively) (Fig. S1, Tables S1-S3). For each embryo, temporal age was noted (hAEL), as well as its developmental stage defined by the number of abdominal segments expressing *inv*.

Measurements were processed and analyzed using custom Python (Van Rossum, 1995) and R (R. Development Core Team, 2008) scripts. Pairwise comparisons were performed using one-way ANOVA and corrected for multiple testing (per dimension measured, e.g. growth zone width) with the Holm procedure (Holm, 1979).

### Segmentation rate

Segmentation rate was assessed for a deviation from linear using a separately collected specifically designated sample of 123 embryos. Embryos from multiple clutches were collected over the space of 1 h, then randomly assigned a time window (ranging from 44-55 h after collection) and fixed at this time. The collection thus generated contains embryos of ages randomly spread within each 60 min time window. From this data set, nine embryos were sampled randomly in each time window to generate a total sample of 108 embryos that can be assumed to be a uniformly distributed sample within the total age range of 44-56 hAEL. If any stage of segmentation is shorter or longer than the others, then it should be overrepresented or underrepresented in this uniform sample (when excluding stages 1 and 9, as we cannot assume they start exactly at 44 h and end exactly at 56 h, respectively). This was tested using a Chi-squared goodness of fit test using the R platform.

### Visualization of cell division

Fluorescent images were acquired with an Olympus FV1200 spectral confocal system based on an IX-83 inverted microscope stand, using a 40×/NA=0.95 air objective. Confocal fluorescence images of DAPI and Alexa 488 anti-PH3 and non-confocal transmitted light DIC images were acquired. The DAPI channel used 405 nm excitation and a 430 nm low-pass emission band, and the Alexa 488 channel used 488 nm excitation and a 505 nm low-pass emission band, acquired sequentially. *z*-stacks were acquired with 1.5 µm spacing. Fields were tiled with no overlap in order to acquire large areas. Stacks were collapsed to a single image using a maximum intensity *z*-projection.

Combined *in situ* and antibody labeling images were created by filtering out the BM-Purple staining only and converting it to red pseudocolor using Adobe Photoshop, and combining that as a separate channel with the DAPI and PH3 channels.

For averaging cell division patterns, 35 images of different embryos from mixed stages (in the range of 46-54 hAEL; note that the exact stage cannot be determined based on morphology alone) were aligned on the widest part of the growth zone. These images were subsequently averaged in Fiji and subjected to a Gaussian blur with σ=1 µm to generate a heat map of cell division in the growth zone.

### Quantification of cell division

The total number of Alexa 488 anti-PH3-labeled cells and the total number of DAPI-stained cells were calculated using the Fiji distribution of ImageJ (Schindelin et al., 2012; Schneider et al., 2012) and the 3D Droplet Finder plugin (http://imagejdocu.tudor.lu/doku.php?id=plugin:analysis:droplet_counter:start). Briefly, the raw images were leveled using a rolling-ball filter (radius=50 pixels), blurred (Gaussian blur, σ=1.75 µm), and then run through the Droplet Finder plugin set. A Python script was written to convert the output of the Droplet Finder plugin to a set of point regions of interest. This enabled us to mark the detected nuclei and visualize them with the 3D-viewer plugin. In a crowded 3D field, the cells could not be manually counted, so we cannot provide a quantitative comparison to ʻground truth'. However, the segmentation was consistent with qualitative observation of the marked nuclei. For the more sparsely labeled Alexa 488 PH3 images, the automated detection was within 91.3±15.2% of ground truth when compared with human observation.

We note that DAPI staining is not maintained throughout mitosis, and as such the total number of cells counted by DAPI-stained nuclei is underestimated. Given that the proportion of cells in mitosis is already low (2-5%), the underestimation of the total number of cells falls within the range of error and was not further corrected.

The procedure was applied to three different areas in the growth zone, which were distinguished by the expression pattern of *eve* (Fig. 6B). This analysis was performed on a sample of 45 embryos stained for PH3 and *eve* RNA, separated into different 2 h age groups, evenly spread out over ages of interest from 44-56 hAEL, as well as 23 additional embryos for which age was unknown (Fig S3, Table S4). One-way ANOVA was performed in R on the ratio of PH3^+^ to DAPI-stained cells, comparing the proportion of cells in mitosis in the different areas of the growth zone, controlled for individual embryos, and corrected for multiple testing with the Holm procedure (Holm, 1979).
